# Differential Effects of Histone Acetyltransferase GCN5 or PCAF Knockdown on Urothelial Carcinoma Cells

**DOI:** 10.3390/ijms18071449

**Published:** 2017-07-05

**Authors:** Evangelia A. Koutsogiannouli, Nicholas Wagner, Christiane Hader, Maria Pinkerneil, Michèle J. Hoffmann, Wolfgang A. Schulz

**Affiliations:** Department of Urology, Heinrich Heine University, 40225 Düsseldorf, Germany; evkouts@gmail.com (E.A.K.); nicholas.wagner@hhu.de (N.W.); christiane.hader@hhu.de (C.H.); maria.lehmann@hhu.de (M.P.); michele.hoffmann@hhu.de (M.J.H.)

**Keywords:** histone acetyltransferases, urothelial carcinoma, bladder cancer, GCN5, PCAF, p300, CBP, MDM2, c-MYC, epigenetic drugs

## Abstract

Disturbances in histone acetyltransferases (HATs) are common in cancers. In urothelial carcinoma (UC), p300 and CBP are often mutated, whereas the GNAT family HATs GCN5 and PCAF (General Control Nonderepressible 5, p300/CBP-Associated Factor) are often upregulated. Here, we explored the effects of specific siRNA-mediated knockdown of GCN5, PCAF or both in four UC cell lines (UCCs). Expression of various HATs and marker proteins was measured by qRT-PCR and western blot. Cellular effects of knockdowns were analyzed by flow cytometry and ATP-, caspase-, and colony forming-assays. GCN5 was regularly upregulated in UCCs, whereas PCAF was variable. Knockdown of GCN5 or both GNATs, but not of PCAF alone, diminished viability and inhibited clonogenic growth in 2/4 UCCs, inducing cell cycle changes and caspase-3/7 activity. PCAF knockdown elicited GCN5 mRNA upregulation. Double knockdown increased c-MYC and MDM2 (Mouse Double Minute 2) in most cell lines. In conclusion, GCN5 upregulation is especially common in UCCs. GCN5 knockdown impeded growth of specific UCCs, whereas PCAF knockdown elicited minor effects. The limited sensitivity towards GNAT knockdown and its variation between the cell lines might be due to compensatory effects including HAT, c-MYC and MDM2 upregulation. Our results predict that developing drugs targeting individual HATs for UC treatment may be challenging.

## 1. Introduction

Bladder cancer is the fifth most common cancer in the Western world, with about 400,000 new diagnosed cases per year and 150,000 deaths worldwide [1]. It is the third most common cause of cancer deaths among Western men, with large variations in its course due to its unpredictable biological behavior and response to treatment [2]. Important exogenous risk factors for bladder carcinogenesis are smoking, environmental and occupational exposure to chemical carcinogens and dietary factors [3]. Urothelial carcinoma (UC) is the most common type of bladder cancer representing 90% of cases in industrialized countries. UC is a highly heterogeneous disease including superficial papillary tumors, which tend to recur, but are generally not life-threatening, and muscle-invasive carcinomas, with a much higher death risk due to distant metastasis and local complications. However, up to 70% of superficial cases suffer recurrences and more than 10% progress to invasive UC [2].

According to the Cancer Genome Atlas Research Network and concurrent reports by others, genes encoding chromatin regulators are more frequently mutated in urothelial carcinoma than in other common cancers [4,5]. Among the mutated chromatin regulators are several histone acetyltransferases (HATs), especially CBP (Gene *KAT3A*, formerly *CREBBP*) and p300 (*KAT3B*, formerly *EP300*).

HATs catalyze the acetylation of conserved lysine (K) residues on histones and non-histone substrates using acetyl-CoA as the acetyl group donor; this process can be reversed by histone deacetylases (HDACs). HATs are classified into two distinct groups based on their cellular location and mechanism of catalysis: the nuclear A-type HATs with direct impact on gene transcription, and the cytoplasmic B-type HATs which catalyze the acetylation of newly synthesized histones H3 and H4 [6]. The GNAT (GCN5-related *N*-acetyltransferase) family members GCN5 and PCAF (General Control Nonderepressible 5, p300/CBP-Associated Factor; genes: *KAT2A* and *KAT2B*) belong to the A-type HATs. A-type HATs are less diverse in domain organization than the others. They form multiprotein complexes with diverse biological functions including global and locus-specific histone acetylation and acetylation of non-histone proteins [7]. It is thought that GCN5 and PCAF perform many redundant, but also some specific functions. Both can act as components of the ATAC (Ada2a-containing) and STAGA (SPT3-TAF9-GCN5 acetylase) complexes, which share some subunits but differ in others [8]. The composition and the precise functions of these complexes are still not completely characterized. In various cancers, GCN5 acts in a rather oncogenic fashion [9,10,11,12], which is usually ascribed to its function as a coactivator of the c-MYC (V-Myc Avian Myelocytomatosis Viral Oncogene Homolog) oncogene protein [13,14,15]. For PCAF, both pro- and anti-tumorigenic properties have been observed in different cancer types [16,17,18,19,20,21]. Specifically, PCAF regulates MDM2 (Mouse Double Minute 2) stability and function, and vice versa [22,23,24].

Here, we report our findings on the expression of PCAF and GCN5 as well as the effects of their small interfering ribonucleic acid (siRNA)-mediated downregulation (“knockdown”) on growth and survival in urothelial carcinoma cell lines.

## 2. Results

### 2.1. Expression of HATs in Urothelial Carcinoma Cell Lines and Non-Cancerous Cells

We first performed a database search on genomic alterations of the histone acetyltransferases GCN5 (*KAT2A*), PCAF (*KAT2B*), CBP (*KAT3A*), p300 (*KAT3B*), MYST3 (*KAT6A*), MYST4 (*KAT6B*), and TRRAP (*TRRAP*), an adapter protein found in various multiprotein chromatin complexes with HAT activity, in UC tissues and cell lines. Data generated by The Cancer Genome Atlas (TCGA) Research Network (Available on: http://cancergenome.nih.gov/) was searched via cBioPortal [25] and COSMIC (Catalogue of somatic mutations in cancer) [26]. p300 was the most frequently mutated HAT followed by its paralog CBP. The search moreover revealed that in UC tissues, p300 and CBP are often affected by deletions, missense mutations or truncating mutations, whereas GCN5, PCAF, MYST3 are rather amplified or upregulated, and MYST4 shows the same but fewer alterations. TRRAP was likewise mutated in some UC tissues (Figure S1).

As a prerequisite for mechanistic investigations, the expression of six HATs, namely GCN5, PCAF, p300, CBP, MYST3 and MYST4, as well as TRRAP was measured by qRT-PCR in a large series of UC cell lines (UCCs), which represent different stages and grades across the disease. For comparison, several independent cultures of normal urothelial cells (UP) were used.

**Figure 1:**
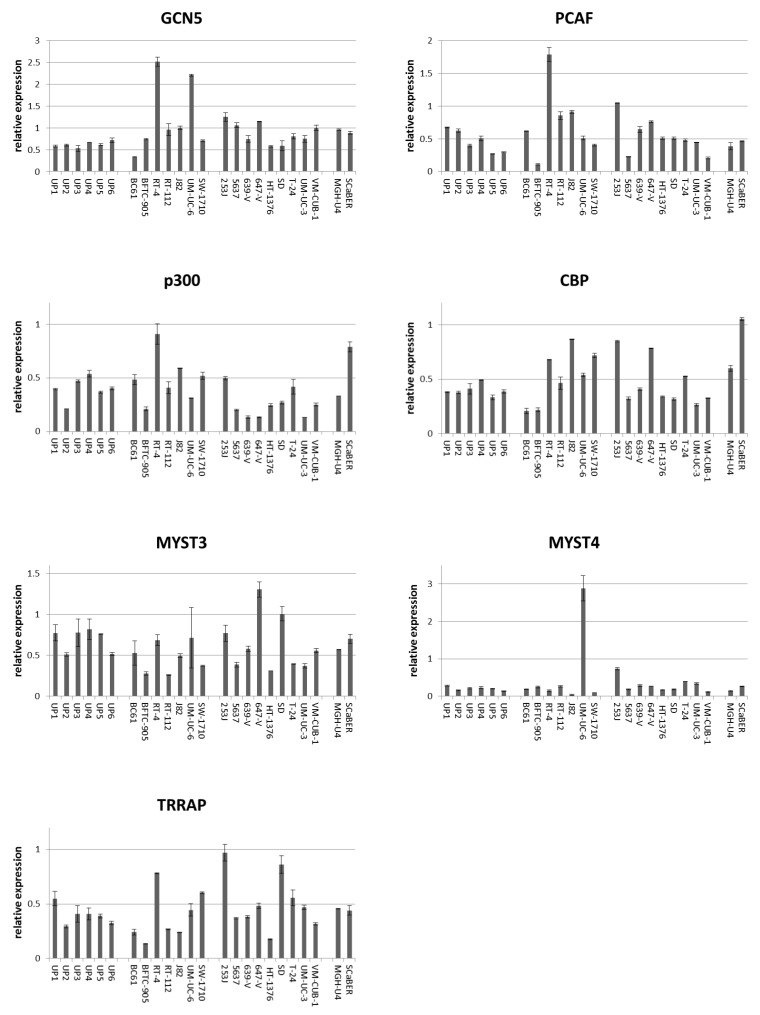
Expression of mRNAs of six histone acetyltransferases (HATs) and TRRAP in normal urothelial cells (UP) and 18 urothelial cancer cell lines. mRNA levels were quantified by qRT-PCR with TBP mRNA as a reference for (**A**) PCAF, (**B**) GCN5, (**C**) p300, (**D**) CBP, (**E**) MYST3, (**F**) MYST4, and (**G**) TRRAP in 6 independent cultures of normal urothelial cells and 18 urothelial carcinoma cell lines (UCCs). Note the low expression of PCAF in BFTC-905, low expression of p300 in 639-V, 647-V and UM-UC-3, and high expression of MYST4 in UM-UC-6. Characteristics of the UCCs are summarized in Table S1.

According to qRT-PCR, GCN5 mRNA was at least as strongly expressed as in UP cultures in all UCCs but BC61, which had been established from a pTaG2 papillary tumor. By comparison, PCAF expression was variable, with some UCCs exhibiting lower and others higher mRNA levels compared to the range seen in UP cultures (Figure 1). PCAF expression was particularly low in BFTC-905 originating from a high-grade papillary tumor. Expression of p300 and CBP mRNA was variable in UCCs and UPs, but p300 mRNA was distinctly decreased in individual cell lines known to harbor inactivating mutations (especially 639-V and 647-V). Similarly, no systematic overall differences between UCCs and UPs were observed between normal and cancerous cells for MYST3, MYST4 and TRRAP, although individual cell lines displayed conspicuously high or low expression, e.g., UM-UC-6 for MYST4 (Figure 1).

**Figure 2:**
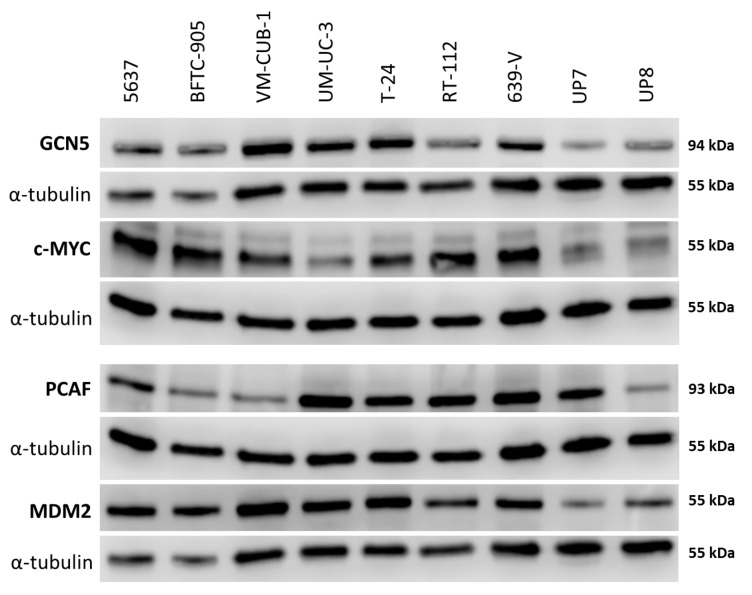
Protein levels of GCN5 and PCAF in cancer cell lines and normal cells. Western blot analysis of PCAF and GCN5 expression in the indicated urothelial carcinoma (UC) cell lines and two normal urothelial cell cultures (UP7, UP8). In addition, their potential interactors MDM2 and c-MYC were analyzed, respectively. MDM2 (Mouse Double Minute 2) was detected on the same membrane as GCN5 after stripping; c-MYC was detected on the same membrane as PCAF after stripping. α-Tubulin was used as a loading control once for each membrane and is shown beneath each of the proteins of interest. A quantification of the signals is illustrated in Figure S2 and summarized in Table S1.

Western blot analyses confirmed the tendency towards generally increased expression of GCN5 in UCCs as well as the variable expression of PCAF predicted by the qRT-PCR measurements (Figure 2). Adjusted to α-tubulin as a reference, GCN5 protein was up to 5 times more abundant in UCCs than in UPs (Figure S2A). Notably, whereas PCAF protein was increased in some UCCs, its expression was similar to that in UPs in individual UCCs (Figure 2 and Figure S2B) and was again particularly low in BFTC-905.

Both MDM2 and c-MYC protein expression was generally stronger in UCCs than in UPs (Figure 2 and Figure S2C,D). No significant positive or negative correlation between the expression of either PCAF and MDM2 or GCN5 and c-MYC could be discerned.

Based on these results (Table S1), we decided to focus the present study on the GNAT family HATs GCN5 and PCAF.

### 2.2. Effects of GCN5 and PCAF Single Knockdown

**Figure 3:**
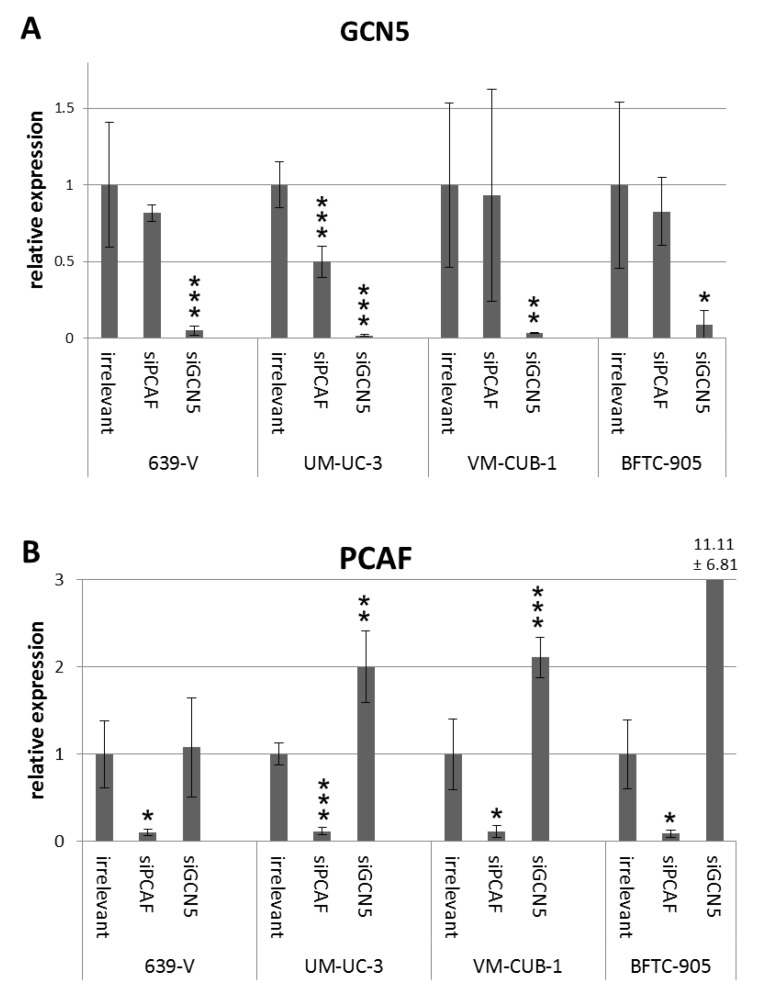
GCN5 (**A**) and PCAF (**B**) mRNA levels after siRNA treatment. GCN5 and PCAF mRNA was quantified in four cell lines (639-V, UM-UC-3, VM-CUB-1, BFTC-905) treated with siRNA against PCAF, GCN5 or a non-targeting control (irrelevant) by qRT-PCR with TBP as a reference. Asterisks denote significant differences, * *p* < 0.05, ** *p* < 0.01, *** *p* < 0.001. Note the clear compensatory increase in PCAF mRNA following GCN5 knockdown in most cell lines and the tendency towards decreased GCN5 expression after PCAF downregulation.

To investigate the involvement of GCN5 and PCAF in cell proliferation, siRNAs designed to downregulate GCN5 or PCAF or irrelevant (non-targeting) siRNA were transiently transfected into the UCCs VM-CUB-1 and BFTC-905 (epithelial phenotype) as well as into 639-V and UM-UC-3 (mesenchymal phenotype). These cell lines were selected from the larger panel for their different expression levels of PCAF and GCN5 (Figure 1 and Figure 2). 639-V and UM-UC-3 have high PCAF protein expressions whereas VM-CUB-1 and BFTC-905 have lower protein expressions, similar to non-cancerous cells. For GCN5, expression was highest in VM-CUB-1 followed by 639-V and UM-UC-3, and was lowest in BFTC-905 and normal urothelial cells. In all UCCs, efficient knockdown of the targeted mRNAs and proteins was achieved at 72 h after transfection as shown by qRT-PCR analysis and western blotting (Figure 3 and Figure S3). Of note, knockdown of GCN5 consistently led to upregulation of PCAF mRNA in UM-UC-3, VM-CUB-1 and BFTC-905 (Figure 3B). Knockdown of PCAF led instead to a moderate downregulation of GCN5 in 639-V and UM-UC-3 as well (Figure 3A). These changes were, however, only partly reflected on the protein level (Figure S3).

**Figure 4:**
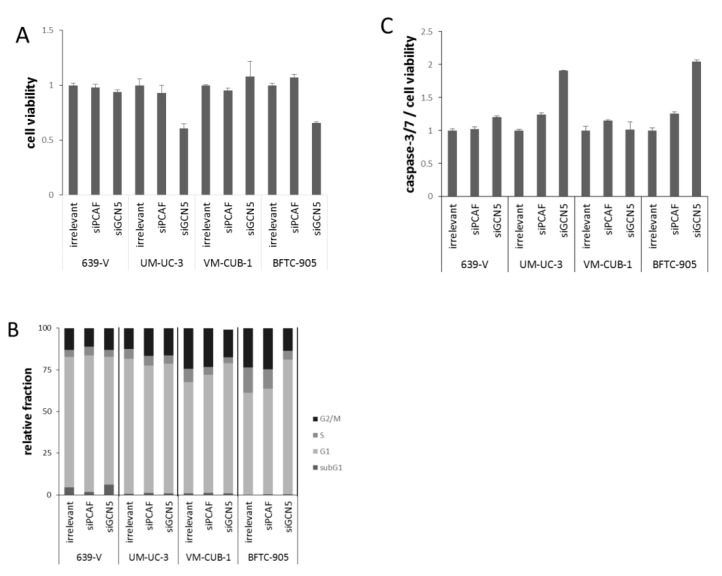
Cellular effects of single GNAT knockdown. (**A**) Cell viability 72 h after GCN5 or PCAF knockdown in UCCs as measured by CellTiter-Glo assay (measuring total ATP). For each cell line, values were set as 1 for the treatment with irrelevant siRNA; (**B**) Cell cycle distribution after GCN5 or PCAF knockdown in UCCs as measured by flow cytometry (mean of triplicate values). The amount of apoptotic cells is reflected as subG1 fraction. Note an increased G2/M fraction in UM-UC-3, a slight increase in the G1 fraction in VM-CUB-1 and a more profound G1 arrest in BFTC-905 cells treated with siGCN5; (**C**) Caspase 3/7 activity after GCN5 or PCAF knockdown in UCCs. Values obtained by the Caspase-Glo assay were normalized to viability of each condition. For each cell line, values were set as 1 for the treatment with irrelevant siRNA.

**Figure 5:**
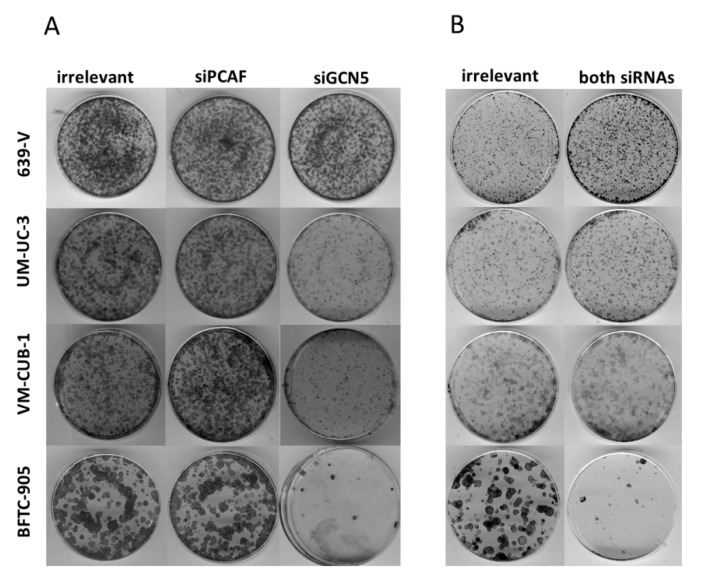
Effects of GNAT siRNA treatment on UCC clonogenicity. Colony formation assays after treatment of the indicated UCCs with siRNAs against GCN5, PCAF (**A**) or both HATs (**B**). Irrelevant designates treatment with non-targeting siRNA. Representative plates from triplicate experiments are shown.

At 72 h after GCN5 knockdown by siRNA transfection, the number of viable cells was strongly diminished in BFTC-905 and UM-UC-3 cells, but not in the two other cell lines (Figure 4A). GCN5 knockdown also inhibited clonogenic growth almost completely in BFTC-905, and less strongly in UM-UC-3 and VM-CUB-1 (Figure 5). In contrast, knockdown of PCAF did not affect viability after 72 h, or clonogenicity in any cell line (Figure 4A and Figure 5).

To further characterize the impact of PCAF or GCN5 knockdown, cell-cycle distribution and caspase-3/7 activity were analyzed in all cell lines (Figure 4B,C). Overall, only slight differences were detected by flow cytometry in the cell cycle distribution of cells treated with GCN5 or PCAF siRNAs compared to the irrelevant siRNA. In UM-UC-3, a slight increase in the G2/M phase was observed upon treatment with either siRNA (Figure 4B), whereas in VM-CUB-1, the G1 fraction was slightly increased. The only major effect was an evident G1 arrest in BFTC-905 treated with GCN5 siRNA, whereas PCAF siRNA had little effect (Figure 4B). Significant increases in the subG1-fraction were detected in none of the cell lines and with neither GCN5 nor PCAF siRNA, even in BFTC-905 cells treated with GCN5 siRNA. However, the more sensitive caspase-3/7 assay revealed slightly increased apoptosis in BFTC-905 and UM-UC-3 following GCN5 knockdown (Figure 4C), in accord with the significant decrease in cell viability.

### 2.3. Effects of GCN5 and PCAF Double Knockdown

**Figure 6:**
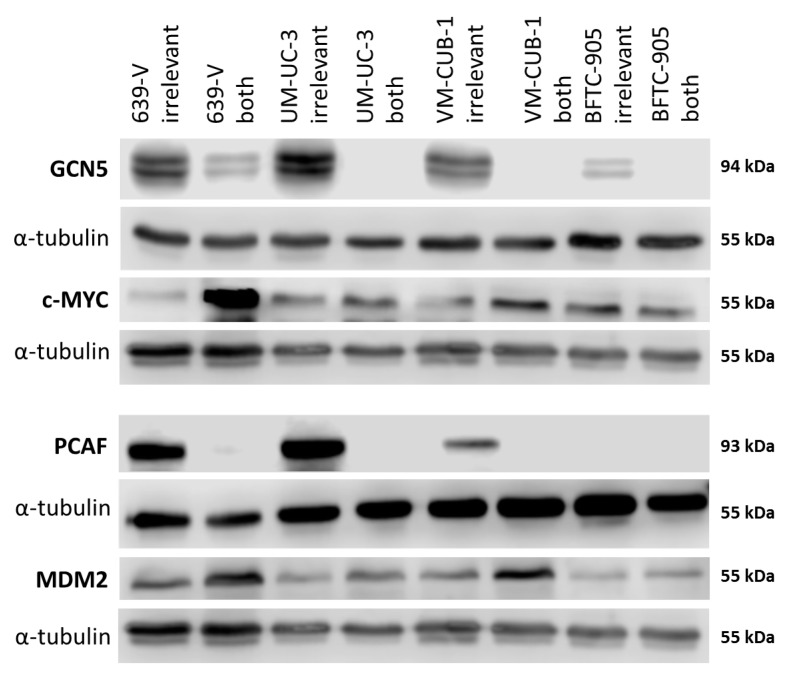
Protein levels of GCN5 and PCAF and potentially interacting proteins in UCCs after combined knockdown. Western blot analysis of PCAF and GCN5 expression in the indicated UC cell lines following treatment with non-targeting (irrelevant) siRNA or with siRNAs against PCAF and GCN5 (both). In addition, their potential interactors MDM2 and c-MYC were analyzed, respectively. α-Tubulin was used as a loading control c-MYC was detected on the same membrane as MDM2 after stripping. α-Tubulin was used as a loading control once for this blot and is shown beneath each of the proteins of interest. A quantification of the signals is illustrated in Figure S4.

**Figure 7:**
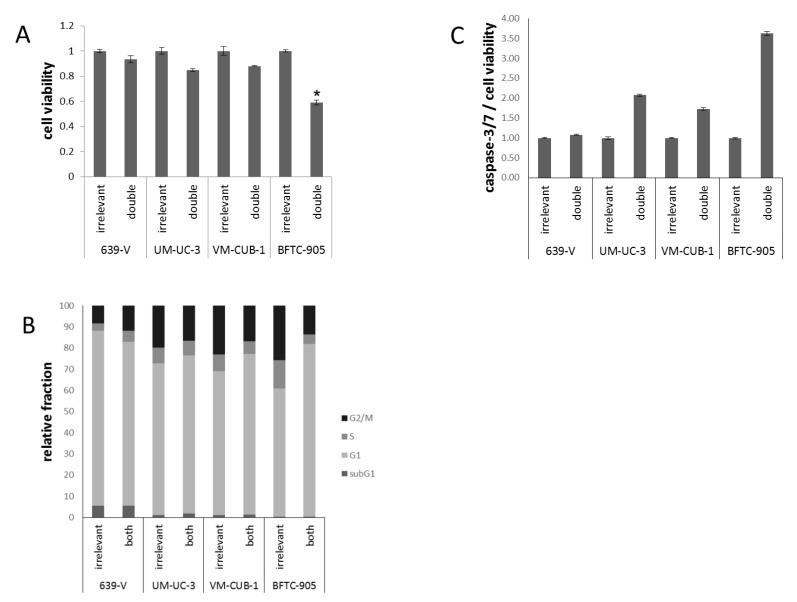
Cellular effects of double GNAT knockdown. (**A**) Cell viability 72 h after combined GCN5/PCAF knockdown in UCCs as measured by CellTiter-Glo assay (measuring total ATP). For each cell line, values were set as 1 for the treatment with irrelevant siRNA; (**B**) Cell cycle distribution after combined GCN5/PCAF knockdown in UCCs as measured by flow cytometry (mean of triplicate values). The amount of apoptotic cells is reflected as subG1 fraction; (**C**) Caspase 3/7 activity after GCN5 or PCAF knockdown in UCCs. Values obtained by the Caspase-Glo assay were normalized to viability of each condition. For each cell line, values were set as 1 for the treatment with irrelevant siRNA. Asterisks denote significant differences, * *p* < 0.05.

Because of the limited effects of single knockdown, and a hint at compensatory upregulation of PCAF following GCN5 knockdown (Figure 3), the four UCCs were double-transfected with siRNAs to downregulate both GNATs. In all UCCs, efficient knockdown of both proteins was achieved 72 h after transfection as shown by western blot analysis (Figure 6 and Figure S4A,B). Nevertheless, at 72 h after siRNA transfection, the number of viable cells was significantly diminished only in BFTC-905, to a lesser degree in UM-UC-3 and, different to GCN5 single knockdown, also in VM-CUB-1 (Figure 7A). Similarly, clonogenic growth in BFTC-905 was almost completely inhibited. However, UM-UC-3 and VM-CUB-1 retained their ability to form colonies even though both HATs were downregulated (Figure 5B). This result is in notable contrast to the effect of GCN5 downregulation alone, which impeded their potential to form colonies. Clonogenicity of 639-V was not diminished by the double knockdown.

Flow cytometry (Figure 7B) revealed slight differences in cell cycle distribution in double-transfected cells, as compared to cells treated with control siRNA. In 639-V, a slight increase in the G2/M fraction was recorded, whereas for the other cell lines the G1 fraction increased. Again, the strongest G1 increase was seen in BFTC-905 cells. No significant elevation of the subG1-fraction could be detected. Moderately elevated caspase-3/7 activity was found in UM-UC-3 and BFTC-905 cells after double knockdown (Figure 7C), comparable to the effects of single GCN5 knockdown. VM-CUB-1 showed elevated caspase-3/7 activity after double knockdown, in contrast to single GCN5 knockdown.

Since following transfection with siGCN5 or both GCN5 and PCAF siRNAs, BFTC-905 cells became arrested in G1 and largely lost the ability to form clones, but underwent apoptosis only to a limited degree, we tested whether the treatment induced senescence by staining for the biomarker senescence-associated β-galactosidase (SA-βgal). However, the number of senescent cells did not differ significantly between control and transfected cells (Figure S5).

The most stringent assay for detecting malignant transformation of cells in vitro is testing for their ability to form colonies in soft agar. Therefore, following GNAT knockdown, soft agar assays were performed for 639-V and UM-UC-3, since VM-CUB-1 and BFTC-905 do not readily form colonies in soft agar. For both cell lines two cell densities (10,000 and 50,000 cells/well) were seeded. In all cases, colonies of transformed cells were formed at a comparable number to the control samples (Figure S6).

### 2.4. Effects of GCN5 and PCAF Double Knockdown on Expression of Potential Interactors

**Figure 8:**
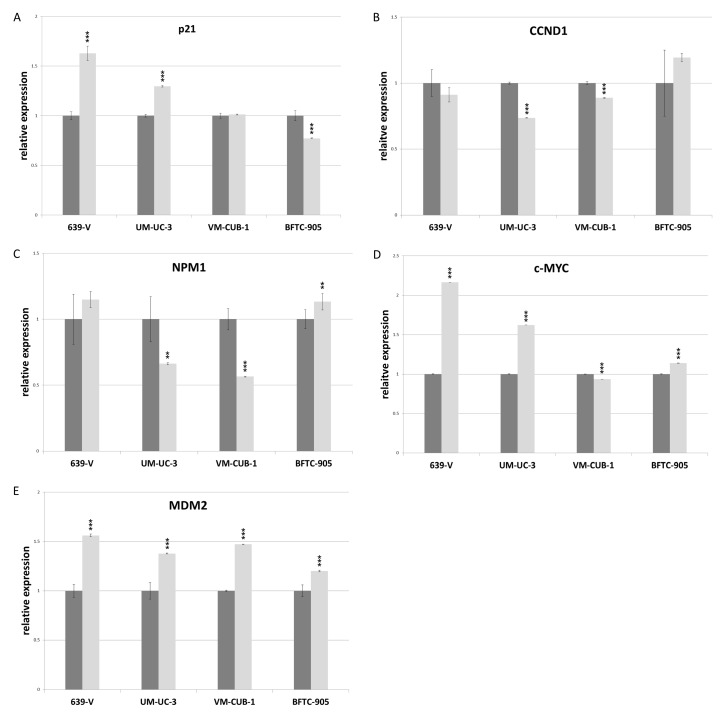
RNA levels of p21 (**A**), CCND1 (**B**), NPM1 (**C**), c-MYC (**D**) and MDM2 (**E**) after double knockdown of GCN5 and PCAF in 639-V, UM-UC-3, VM-CUB-1 and BFTC-905 (from left to right). All values were determined by qRT-PCR, adjusted to TBP mRNA as a reference. Dark bars: irrelevant siRNA (values set as 1 for each cell line); light bars: GCN5 + PCAF siRNA. Asterisks denote significant differences, ** *p* < 0.01, *** *p* < 0.001.

The CDK inhibitor p21 (gene: *CDKN1A*) and the cell cycle driver Cyclin D1 (gene: *CCND1*) often respond sensitively to changes in epigenetic regulation, for example to inhibition of histone deacetylases. We measured expression of these two inhibitors by qRT-PCR following GNAT knockdown, but no consistent changes were observed apart from slight upregulation of p21 in 639-V and UM-UC-3 cells (Figure 8A,B). Expression of a general c-MYC target gene, nucleophosmin (gene: *NPM1*) was not significantly affected by GCN5 and PCAF double knockdown (Figure 8C). Interestingly, similar to p21, c-MYC protein was upregulated in 639-V and UM-UC-3 cells, but not in VM-CUB-1 and BFTC-905 following double knockdown (Figure 6 and Figure S4C). These changes were partly reflected at the mRNA level (Figure 8D). In a similar fashion, MDM2 protein was upregulated following GCN5 and PCAF double knockdown in all cell lines, but least so in BFTC-905 (Figure 6 and Figure S4D). This change was slight, but detectable at the mRNA level (Figure 8E).

## 3. Discussion

Despite the discovery of prevalent mutations in various histone acetyltransferase (HAT) genes in urothelial carcinoma, the function of these enzymes is poorly studied in this cancer type. Thus, the present investigation on the GNAT family HATs GCN5 and PCAF is—to our knowledge—the first in bladder cancer. Our findings are in good accord with those in other cancer types, which suggest general protumorigenic functions for GCN5, but not for PCAF [8,9,10,11,12,16,17,18,19,20]. According to database analyses, GCN5 is overexpressed in many UC tissues and—as shown here—in UC cell lines. Efficient GCN5 knockdown inhibited short-term and long-term proliferation of specific, albeit not all UCCs. In the sensitive cell lines, knockdown resulted in G1 cell cycle arrest, albeit not senescence, and moderately increased caspase-3/7 activity indicative of a moderate degree of apoptosis. The limited impact of the efficient knockdown might partly result from upregulation of PCAF in response to GCN5 knockdown, which may act in a compensatory fashion. Interestingly, PCAF knockdown did not elicit an analogous upregulation of GCN5, which was instead slightly downregulated. Although these changes were not fully reflected at the protein level, these observations hint at transcriptional cross regulation between these two HATs. Cross regulation and compensation are common among paralogous chromatin regulators, e.g., between HDAC1 and HDAC2 [27] or UTX/KDM6A and JMJD3/KDM6B [28].

Whereas GCN5 knockdown affected at least two cell lines, PCAF knockdown had remarkably little impact on UCC proliferation and survival. This finding accords again with the concept of GCN5 acting in a more oncogenic, and PCAF in a more tumor-suppressive, manner. However, different from some other cancer types [17,18,20,21], in UC cell lines, no indication of enhanced proliferation or clonogenicity was seen following PCAF downregulation.

Notably, double GNAT knockdown did not exert stronger effects in the sensitive cell lines and—with slight differences—resembled GCN5 single knockdown. Nevertheless, it is remarkable that at least two cell lines tolerated efficient knockdown of the HAT pair without substantial decreases in viability and long term proliferation. We speculate that this resilience may result from various compensatory mechanisms. In addition to potential cross regulation between PCAF and GCN5, which might extend to other HATs, we specifically observed increases in c-MYC and MDM2 during double knockdown. These increases were partly visible at the mRNA level, suggesting that they are caused by a combination of transcriptional and posttranscriptional mechanisms. Ultimately, the expression of typical c-MYC target genes remained essentially unchanged, as did the expression of the CDK inhibitor p21, which typically increases as a consequence of treatment with epigenetic inhibitors. Specifically, the *CDKN1A* gene encoding p21 has been described as a target of PCAF [29]. An interesting question is whether these compensatory mechanisms may hold up over a longer period of knockdown, e.g., in UC cell lines with stable expression of siRNA against GCN5 or with GCN5 knockout.

The most unexpected finding in our study is the strong selectivity of the anti-proliferative effects of GCN5 knockdown for specific cell lines, which does not accord with the concept of GCN5 as a “supervisor in all-inclusive control of vertebrate cell cycle progression” [30]. However, disturbed cell cycle regulation with deficient checkpoints is a characteristic of essentially all urothelial carcinomas [4,31] and may allow to circumvent supervision by GCN5. Interestingly, the most sensitive cell line BFTC-905 expresses PCAF marginally, but also GCN5 at comparably low levels. Evidently, this constitution sensitizes to GCN5 knockdown, likely by creating a dependency on the paralogous enzyme. Again, dependencies of this kind have repeatedly been observed with other epigenetic regulators, e.g., for ARID1A and ARID1B [32], and specifically for the HATs CBP and p300 [33]. Unfortunately, BFTC-905 was the only cell line in our panel with low expression of both enzymes which makes it difficult to explore this relationship further. One experimental approach might be generating BFTC-905 sublines with the restoration of normal GNAT expression. By comparison, VM-CUB-1 also expressed relatively little PCAF, but high GCN5, which may account for its resilience against GCN5 knockdown in short term experiments. Expression levels of GCN5 and PCAF do not straightforwardly explain, however, why UM-UC-3 was more sensitive to GCN5 knockdown than VM-CUB-1 or 639-V. In terms of our explanation outlined above, its sensitivity may relate to insufficient compensatory mechanisms.

Inhibitors targeting HATs are considered as potential drugs for cancer therapy [34,35]. Typically, these compounds inhibit two or more paralogous enzymes like CBP/p300 or PCAF/GCN5 [36]. In urothelial carcinoma, CBP/p300 are often affected by inactivating mutations and are likely to act as tumor suppressors. Thus, in principle, targeting the PCAF/GCN5 GNAT pair should be the better strategy, as especially GCN5 is often overexpressed. Intriguingly, however, we find that especially a UC cell line with low GCN5 as well as PCAF was most sensitive to GCN5 knockdown. By inference, our findings predict that tumors with low expression of both enzymes would be most sensitive to their pharmacological inhibition, but such cases may, in fact, be infrequent.

## 4. Materials and Methods

Many methods were performed essentially as in a previous study on histone deacetylases [27].

### 4.1. Cell Culture and siRNA Transfection

The knockdown experiments were performed in four UCCs reflecting the heterogeneity of urothelial carcinoma, namely BFTC-905, VM-CUB-1, 639-V and UM-UC-3 (in decreasing order of differentiation). Additional cell lines investigated for HAT expression were RT4, BC61, 253J, 647-V MGHU4, T24, Scaber, SD, HT-1376, RT-112, 5637, SW-1710, J82, and UM-UC-6. For all cell lines, short tandem repeat profiling was performed by standard DNA fingerprint analysis. Important characteristics of the UCCs are summarized in Table S1. Primary cultures of normal urothelial cells (abbreviated UP#) were used as controls and were cultured as described previously [37].

For siRNA-mediated knockdown, UCCs were transfected with 5 nM specific siRNAs directed against PCAF, GCN5, or both PCAF/GCN5 (10 nM in total) or a non-targeting siRNA control (referred to as “irrelevant siRNA”) using Lipofectamine RNAi MAX (Life Technologies, Darmstadt, Germany), according to the manufacturer’s protocol. The siRNAs were Silencer^®^ Select validated siRNA (#4390824, GCN5: s5658; PCAF: s16894) or Silencer^®^ Select Negative Control #2 validated siRNA (#4390846). Cells were analyzed after a further 72 h of cultivation.

### 4.2. Viability and Apoptosis Assays

Cell viability was measured 72 h after transfection via total cellular ATP using the CellTiter-Glo^®^ Luminescent Cell Viability Assay (Promega, Mannheim, Germany). Caspase activity was quantified by the caspase-Glo 3/7 assay (Promega) and normalized to cell viability.

### 4.3. Colony Forming Assay and Giemsa-Staining

For colony forming assays, cells were plated in 6 cm plates at a density of 500 to 1500 cells per plate, 72 h after siRNA transfection. Colonies formed after 10 to 15 days were fixed in methanol and stained with Giemsa (Merck, Darmstadt, Germany).

### 4.4. Flow Cytometry

Cell cycle analyses of treated cells was performed after 72 h of siRNA-mediated transfection by staining the attached and supernatant cells with PI-buffer containing 50 µg/mL propidium iodide, 0.1% sodium citrate and 0.1% Triton X-100 [27]. Flow cytometry analyses were performed using a Miltenyi MACSQuant^®^ Analyzer and evaluated by the MACSQuantify software (Milteny Biotec, Bergisch Gladbach, Germany).

### 4.5. Senescence Assay

Treated cells were washed twice in PBS and fixed for 5 min in 2% formaldehyde and 0.2% glutaraldehyde. Following two washes with PBS cells were stained overnight at 37 °C with fresh senescence associated β-Gal (SA-β-Gal) staining solution (1 mg/mL X-Gal (5-bromo-4-chloro-3-indolyl-β-d-galacto-pyranoside; Merck), 150 mM NaCl, 2 mM MgCl_2_, 5 mM K_3_Fe(CN)_6_, 5 mM K_4_Fe(CN)_6_). Following another wash with PBS images were taken using a Nikon Eclipse TE2000-S microscope (Nikon, Tokyo, Japan).

### 4.6. RNA Isolation, cDNA Synthesis and Quantitative Real-Time PCR

Total mRNA was isolated using the RNeasy Mini Kit (Qiagen, Hilden, Germany) according to the manufacturer’s protocol. cDNA synthesis was performed using the QuantiTect Reverse Transcription Kit (Qiagen, Hilden, Germany) with an extended incubation time of 30 min at 42 °C. qRT-PCR was performed using the QuantiTect SYBR Green RT-PCR Kit (Qiagen, Hilden, Germany) and self-designed primers (Table S2) on the Lightcycler 96 instrument (Roche, Mannheim, Germany). All values were adjusted to *TBP* (TATA-box binding protein) as a reference gene.

### 4.7. Western Blot Analysis

Total protein was extracted by cell lysis for 30 min on ice in RIPA-buffer containing 150 mM NaCl, 1% Triton X-100, 0.5% desoxycholate, 1% Nonidet P-40, 0.1% SDS, 1 mM EDTA, 50 mM TRIS (pH 7.6) and a protease inhibitor cocktail (10 µL/mL, #P-8340, Sigma Aldrich, Munich, Germany). Protein concentrations were determined by BCA protein assay (Thermo Fisher Scientific, Schwerte. Germany). Following SDS-PAGE gel separation and transfer to PVDF membranes (Merck Millipore, Berlin, Germany), membranes were blocked for 1 h with 5% skim milk or bovine serum albumin in TBST (150 mM NaCl, 10 mM TRIS, pH 7.4 and 0.1% Tween-20), and incubated with primary antibodies at room temperature for 1 h or at 4 °C overnight. Antibodies used were Anti-PCAF (C14G9) Rabbit mAb #3378 (Cell Signaling, Danvers, MA, USA), Anti-GCN5L2 (C26A10) Rabbit mAb #3305 (Cell Signaling), Anti-MDM2 Rabbit mAb #OP46 (Oncogene Science, Farmingdale, NY, USA), Anti-c-MYC (D3N8F) Rabbit mAb #13987 (Cell Signaling, Danvers, MA, USA), Anti-α-tubulin Rabbit pAb #4074 (Abcam, Cambridge, UK). After washing, the membranes were incubated with horseradish peroxidase-conjugated secondary anti-rabbit antibody (1:10,000) at RT for 1 h. Bands were visualized by Super Signal™ West Femto (Thermo Fisher Scientific) using a C-DiGit scanner (LI-COR, Bad Homburg, Germany). Protein amounts were calculated from the pixels recorded using Image Studio Digits 4.0 software (LI-COR) relative to α-tubulin as a reference. For detection of multiple proteins on one blot, membranes were incubated in mild stripping buffer (1.5% glycine-HCl, pH 2.2, 0.1% SDS, 1% Tween-20) for 10 min at RT.

### 4.8. Statistical Analysis

Significance of differences between mean values was calculated by paired *t*-test using the GraphPad QuickCalcs Website: https://www.graphpad.com/quickcalcs/ttest1.cfm.

## 5. Conclusions

In conclusion, GCN5 upregulation is especially common in UCCs. GCN5 knockdown impeded growth of specific UCCs, whereas PCAF knockdown elicited minor effects. The limited sensitivity towards knockdown of the two HATs and its variation between the cell lines might be due to compensatory effects including HAT, c-MYC and MDM2 upregulation. Our results predict that developing drugs targeting individual HATs for UC treatment may be challenging.

## Supplementary Materials

Supplementary materials can be found at www.mdpi.com/1422-0067/18/7/1449/s1.

## Abbreviations

c-MYC	V-Myc avian myelocytomatosis viral oncogene homolog
GCN5	General control nonderepressible 5 acetyltransferase
GNAT	GCN5-related *N*-acetyltransferases
MDM2	Mouse double minute 2
PCAF	P300/CBP-associated factor acetyltransferase
UC	Urothelial carcinoma
UCC	Urothelial carcinoma cell line
HDAC	Histone deacetylase
HAT	Histone acetyltransferase
UP	Uroepithel cells, cultured normal urothelial cells
