# Supplementary material for: Differential Effects of Histone Acetyltransferase GCN5 or PCAF Knockdown on Urothelial Carcinoma Cells
Source: Int J Mol Sci. 2017 Jul 5;18(7):1449. doi: 10.3390/ijms18071449 (PMC5535940; doi:10.3390/ijms18071449)
Supplement: Supplementary file 1 [file ijms-18-01449-s001.pdf]

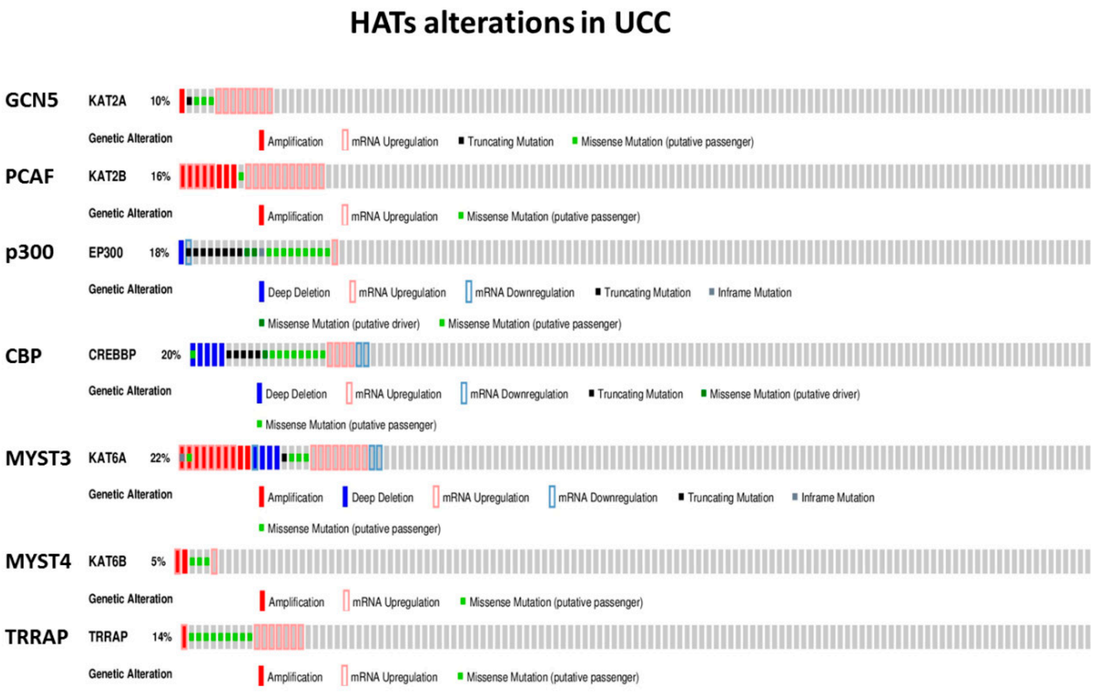

Figure S1. Histone acetyltransferase mutations as recorded by the cBioPortal database (April, 2017).

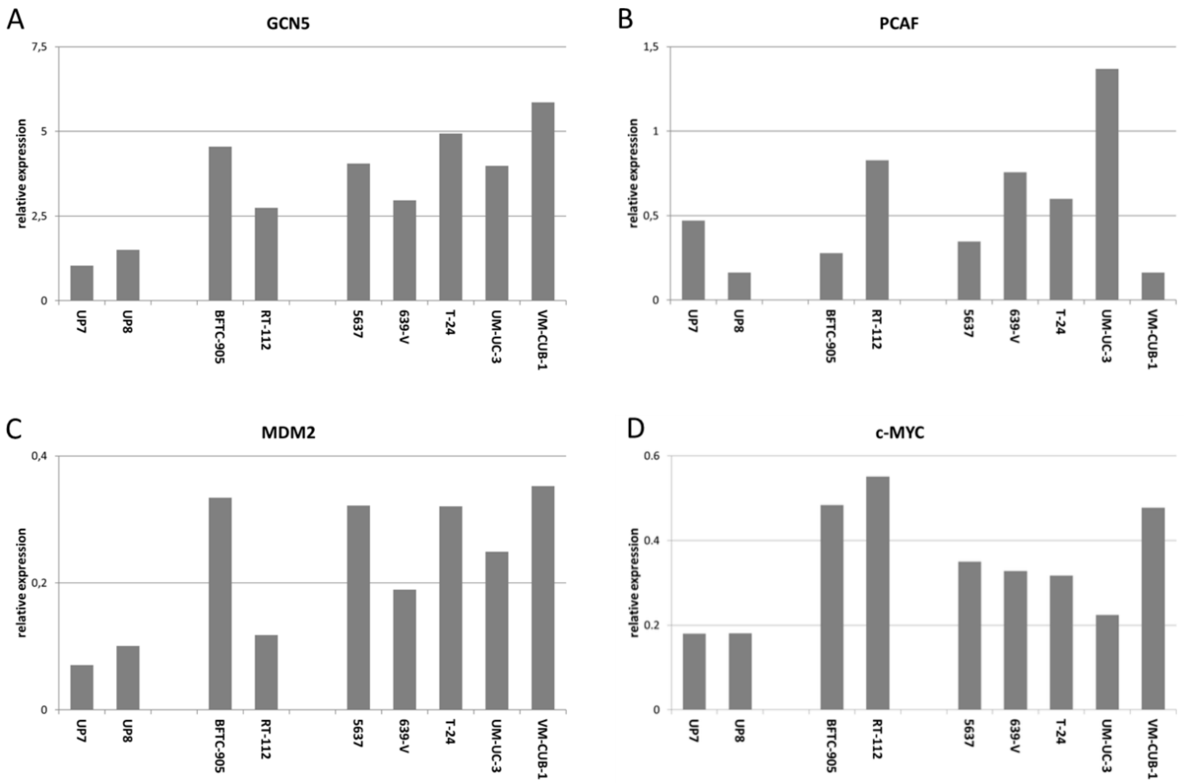

Figure S2. Quantification of western blots in Figure 2.

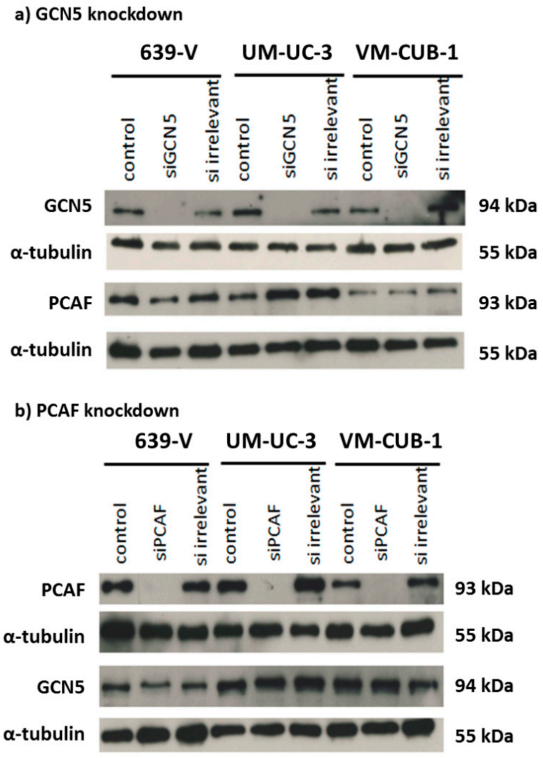

Figure S3. GCN5 and PCAF protein levels after siGCN5 and siPCAF single knockdowns. **(A)** siGCN5 treatment of 639-V, UM-UC-3 and VM-CUB-1. GCN5 was completely knocked down; PCAF expression is increased after GCN5 knockdown in UM-UC-3. Reference α-tubulin blot is shown below. **(B)** siPCAF treatment of 639-V, UM-UC-3 and VM-CUB-1. PCAF was completely knocked down; no severe effect on GCN5 levels was observed. Reference α-tubulin blot is shown below.

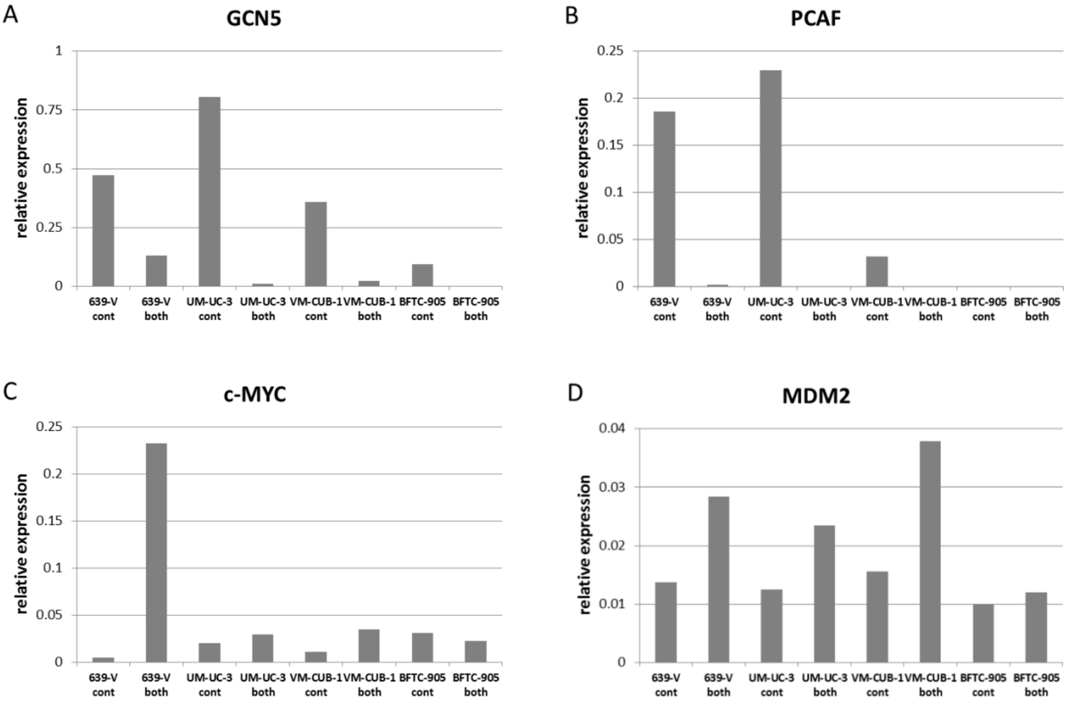

Figure S4. Quantification of western blots in Figure 6 (GCN5/PCAF double knockdown). GCN5 **(A)**, PCAF **(B)**, c-MYC **(C)** and MDM2 **(D)**.

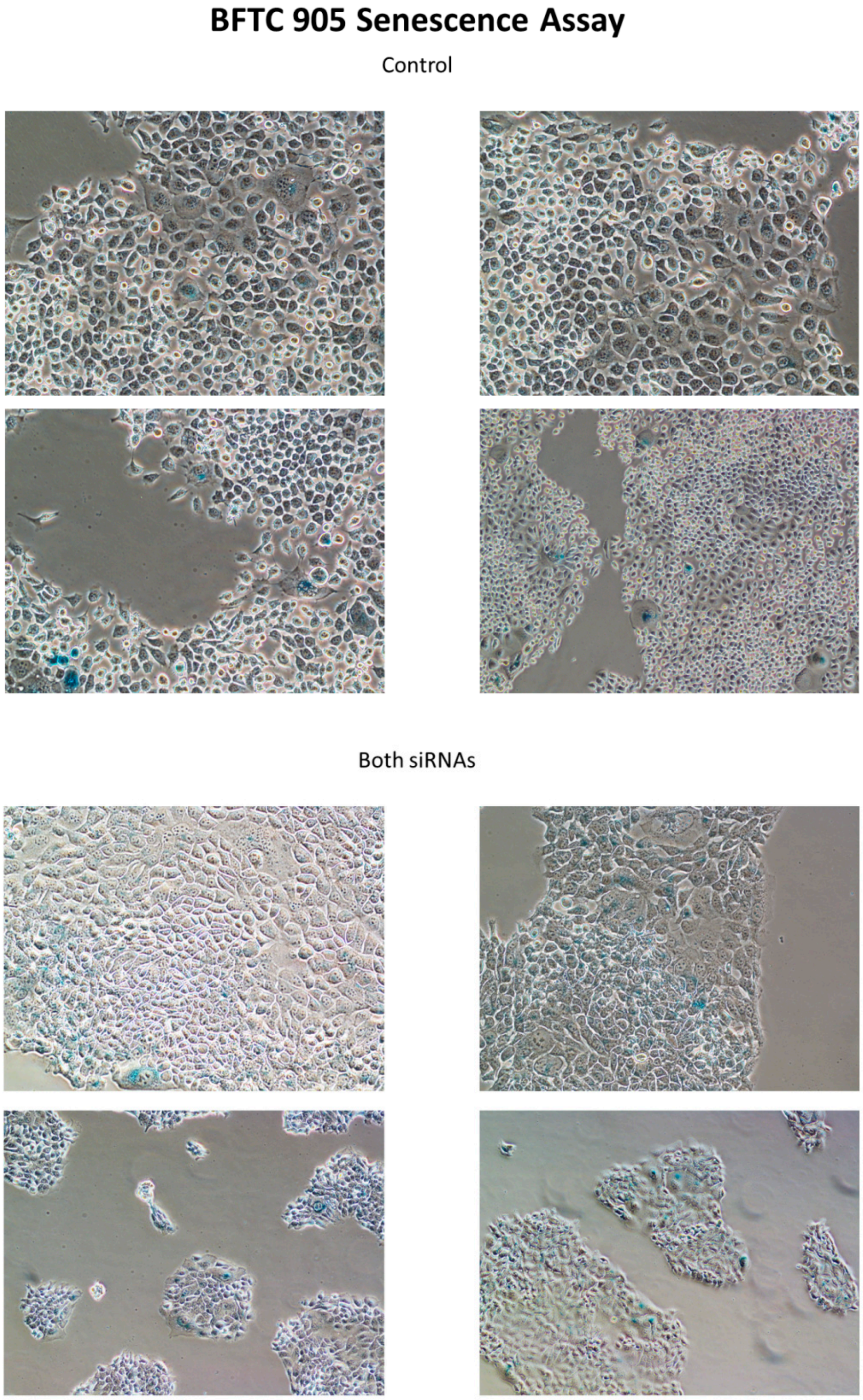

Figure S5. Senescence assay for BFTC-905 after siGCN5/siPCAF double knockdown.

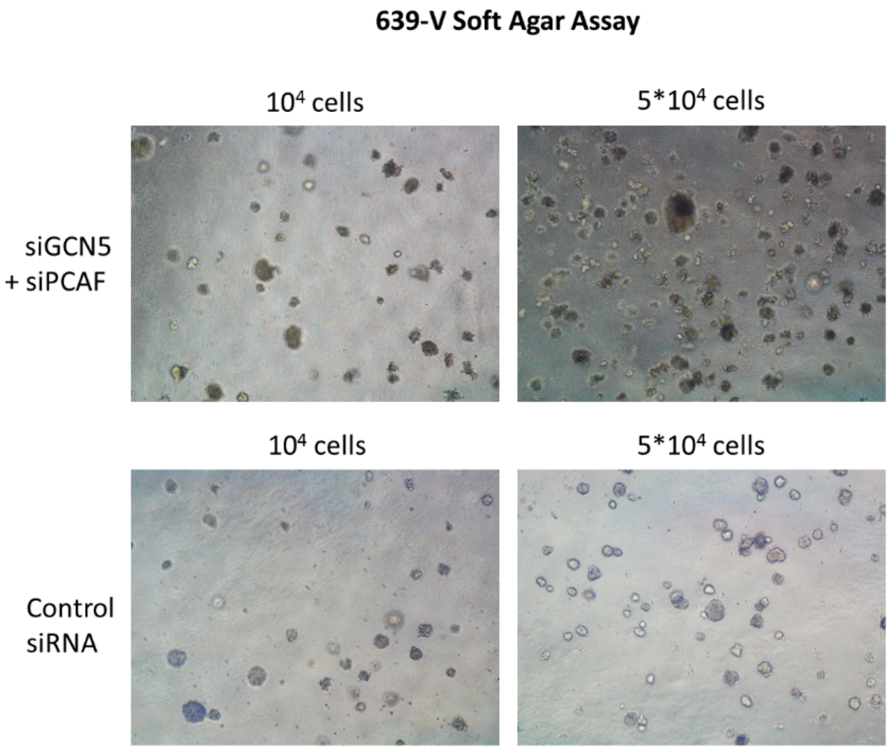

Figure S6. 639-V soft Agar Assay

Table S1. Characteristics of urothelial carcinoma cell lines used

Cell Line	Characteristics of Origin	Sex	Phenotype	GCN5			PCAF		
				mRNA level *	SD	Protein Level §	mRNA Level *	SD	Protein Level §
**5637**	tumor (UC, G2)	M	Epithelial	1.059	0.054	4.0	0.224	0.003	0.3
**BFTC-905**	tumor (UC, G3, papillary)	F	Epithelial	0.743	0.016	4.6	0.017	0.011	0.3
**VM-CUB-1**	tumor (UC)	M	Epithelial	1.004	0.059	5.9	0.206	0.014	0.2
**UM-UC-3**	tumor (UC)	M	Mesenchymal	0.750	0.076	4.0	0.442	0.005	1.4
**T-24**	tumor (UC, G3)	F	Mesenchymal	0.803	0.059	4.9	0.479	0.017	0.6
**RT-112**	tumor (UC, G2, papillary)	F	Epithelial	0.960	0.133	2.7	0.855	0.058	0.8
**639-V**	tumor (UC, G3)	M	Mesenchymal	0.745	0.079	3.0	0.644	0.044	0.8
BC61	UC, pTa, G2	M	Epithelial	0.329	0.012	ND	0.616	0.002	ND
RT-4	UC, G1, T2, papillary	M	Epithelial	2.521	0.104	ND	1.785	0.104	ND
J82	UC, G3, T3	M	Mesenchymal	1.001	0.034	ND	0.912	0.020	ND
UM-UC-6	UC	M	Mesenchymal	2.208	0.023	ND	0.511	0.031	ND
SW-1710	UC, G3, Ta	F	Mesenchymal	0.702	0.020	ND	0.404	0.016	ND
253J	LN metastasis, G4, T4	M	Mesenchymal	1.250	0.095	ND	1.045	0.005	ND
647V	UC, G2	M	Mesenchymal	1.145	0.008	ND	0.760	0.013	ND
HT-1376	UC, G3, >T2	F	Epithelial	0.578	0.013	ND	0.508	0.016	ND
SD	UC		Intermediate	0.591	0.113	ND	0.508	0.015	ND
MGH-U4	UC	M	Intermediate	0.958	0.012	ND	0.387	0.054	ND
SCaBER	Squamous cell carcinoma, G2, T3	M	Epithelial	0.887	0.029	ND	0.461	0.010	ND
**UP7**	normal (primary cells from urethra)	F	Epithelial	0.530	0.067	1.0	0.396	0.019	0.5
**UP8**	normal (primary cells from urethra)	M	Epithelial	0.668	0.002	1.5	0.505	0.036	0.2

* = relative to TBP; § = relative to α-tubulin; ND = not determined; cell lines investigated in detail are marked in bold

Table S2. qPCR Primer sequences.

Gene	Primer name	Sequence
CBP	CBP qPCR Fw	AGTTCCCGTCATCCAGCG
	CBP qPCR Rv	AGGCCCCAGCATGTTGAG
CCND1	CCND1 qPCR fwd	CGCAAACACGCGCAGACCT
	CCND1 qPCR rev	GGAGGGCGGATTGGAAAT
c-MYC	c-MYC qPCR fwd	GCTCCATGAGGAGACACC
	c-MYC qPCR rev	CCTCTTTTCCACAGAAAC
GCN5	GCN5 qPCR Fw	TTCCGAGTGGAGAAGGACA
	GCN5 qPCR Rv	AGCATGGACAGGAATTTGG
MDM2	MDM2 qPCR fwd	CATCGGACTCAGGTACATCTG
	MDM2 qPCR rev	CTTCCTGAAGCTCTTGTACAAGG
MYST3	MOZ qPCR Fw	CCATATCCTCAAGAATACTC
	MOZ qPCR Rv	TGCTGCAGAATAGTTCTAC
MYST4	MYST4 qPCR Fw	AAGGATTTGGACGGTTTCTC
	MYST4 qPCR Rv	CTTCCAATATGCCAGGTAG
NPM1	NPM1 qPCR fwd	GTGGTTCAGGGCCAGTGCA
	NPM1 qPCR rev	CCTTGCTACCACCTCCAGG
p21	p21 qPCR fwd	GGAAGACCATGTGGACCTGT
	p21 qPCR rev	GGCGTTTGGAGTGGTAGAAA
p300	p300 qPCR Fw	GTGCTGGCAACTTACTGAC
	p300 qPCR Rv	ACCATAAGGATTGGGGTTGT
PCAF	PCAF_qPCR_Assay2_Fw	TCAACGAAGACTGCGATCTC
	PCAF_qPCR_Assay2_Rv	GGTTTCGTACCGAGGTAGACTGT
TBP	TBP qPCR fwd	ACAACAGCCTGCCACCTTA
	TBP qPCR rev	GAATAGGCTGTGGGTCAGT
TRRAP	TRRAP qPCR Fw	ACCCTGTCTTTCAGAAGC
	TRRAP qPCR Rv	CCTGGAACGCTGAAGTCA
